# An atlas of novel microtubule-associated proteins in the malaria parasite *Plasmodium falciparum*

**DOI:** 10.1128/mbio.03407-25

**Published:** 2025-12-08

**Authors:** Korbinian Niedermüller, Nick Piwon, Joëlle Paolo Mesen-Ramirez, Leah Zink, Sarah Lemcke, Benjamin Liffner, Cláudia F. Fernandes, Guilherme B. Farias, Arne Alder, Christian Löw, Noa Dahan, Danny W. Wilson, José Cubillán-Marín, Tim W. Gilberger

**Affiliations:** 1 Centre for Structural Systems Biology, Hamburg, Germany; 2 Bernhard Nocht Institute for Tropical Medicine 14888, Hamburg, Germany; 3 University of Hamburg 14915, Hamburg, Germany; 4 University of Adelaide, School of Biological Sciences 110455, Adelaide, South Australia, Australia; 5 Institute for Photonics and Advanced Sensing, University of Adelaide 613297, Adelaide, South Australia, Australia; 6 European Molecular Biology Laboratory, Hamburg Unit 128803 , Hamburg, Germany; University of Geneva, Geneva, Switzerland

**Keywords:** *Plasmodium falciparum*, subpellicular microtubules, microtubule-associated proteins, inner membrane complex, gametocytes

## Abstract

Mature gametocytes of *Plasmodium falciparum* exhibit a characteristic falciform shape, which is conferred by a complex array of subpellicular microtubules (SPMTs) associated with the inner membrane complex (IMC). While several microtubule-associated proteins (MAPs) have been characterized in the related apicomplexan parasite *Toxoplasma gondii*, the identity and function of MAPs in *Plasmodium* remain poorly understood. Here, we employed proximity-dependent biotinylation (BioID) using *Pf*SPM3, a subpellicular MAP, as a bait in order to identify proteins associated with *P. falciparum* gametocyte SPMTs. Mass spectrometry analysis revealed 37 high-confidence proteins in the immediate vicinity of *Pf*SPM3 constituting its “proxiome.” Using GFP tags on the endogenous proteins of 11 highly enriched candidates, we provide a spatial localization atlas of previously uncharacterized proteins. Notably, many of these putative MAPs showed a distinct localization to different structures within the SPMT network, highlighting its complexity in developing gametocytes. Among these, we demonstrate that functional knockout of one MAP, PF3D7_1003400, which exhibits a localization pattern consistent with positioning near the IMC suture/SPMT boundary, interferes with gametocytogenesis and prevents falciform gametocyte development. These findings provide the first atlas of the SPMT network required to coordinate cytoskeletal dynamics, IMC organization, and gametocyte development. Understanding these interactions offers new avenues for blocking gametocyte cytoskeleton development and malaria transmission.

**IMPORTANCE:**

Transmission of *Plasmodium falciparum* relies on the formation of falciform gametocytes, a process dependent on the dynamic interplay between subpellicular microtubules (SPMTs) and the inner membrane complex (IMC). In this study, we employed proximity-dependent biotinylation using *Pf*SPM3, an SPMT-associated protein, as a bait. We present a comprehensive list of highly enriched proteins and a validation of the top candidates revealing novel components of the cytoskeleton of gametocytes that might play a role in their morphogenesis and transmission.

## INTRODUCTION

Of the five *Plasmodium species* that infect humans, *P. falciparum* is responsible for more than 90% of the >590,000 malaria-related deaths recorded annually in tropical and subtropical regions worldwide (1). Malaria parasites have evolved a complex life cycle shuttling between a vertebrate host and a mosquito vector. Despite decades of study, significant gaps remain in our understanding of the cell biology of malaria parasites, hindering the development of novel translational approaches to combat parasite proliferation in the human host and to block its transmission to mosquitoes.

During proliferation within host erythrocytes, about 1–5% of the population is diverted from the asexual multiplication cycle and becomes committed to sexual development (2–4), starting a process called gametocytogenesis. The rate of sexual commitment is influenced by a combination of genetic, epigenetic, and environmental factors, while the duration of gametocytogenesis and its accompanying morphological transitions vary significantly among human-infecting *Plasmodium* species (4). A subgenus of *Plasmodium*, termed *Laverania,* uniquely produces “falciform” or crescent-shaped gametocytes. These falciform gametocytes were first described by Alphonse Laveran during his discovery of malaria parasites in the 19th century, leading to the establishment of the species name “*Plasmodium falciparum*.” In contrast, gametocytes of other human parasites such as *P. vivax* or *P. ovale*, as well as the rodent malaria parasite *P. berghei*, remain round during gametocytogenesis and resemble late stages of the blood stage lifecycle (trophozoites) with some distinguishing features (5–7).

In *P. falciparum*, gametocytogenesis is thought to primarily occur in the bone marrow (8) and progresses through five morphologically distinct stages across ~12 days of development. During this maturation process, the initially round parasite undergoes dramatic elongation, ultimately adopting a characteristic banana-like shape. This transformation is driven by the interplay between the inner membrane complex (IMC, a double membrane structure underneath the parasite plasma membrane, PPM), its associated proteins, and an array of specialized microtubules (MTs). Due to their peripheral position just beneath the PPM and IMC, these MTs are named subpellicular microtubules (SPMTs) (9–11).

Sexually committed ring-stage parasites enter gametocyte development (12–14) with subtle morphological changes that are accompanied by the recruitment of membranous vesicles to nascent IMC plates (15). In blood-stage malaria parasites, MTs are polymerized from a bipartite structure called the centriolar plaque that spans the nuclear envelope (16). The inner centriolar plaque resides within the nucleus and acts as the microtubule organizing center (MTOC) for the intranuclear MTs that coordinate mitosis, while the outer centriolar plaque is exposed to the cytoplasm, acts as the MTOC for the SPMTs, and orients the nascent IMC (17–19). During the transition from stage I to II gametocyte, the SPMTs grow uniformly in both vertical directions, forming a sheet of MTs in stage III (15, 17, 20, 21). This is accompanied by polyglutamylation of the SPMTs, which is likely to be important for their stability (17, 22). By stage IV, the gametocyte reaches its maximal elongation, characterized by pointed ends. At this point, the SPMT network and the IMC are fully developed and support the parasite’s crescent shape. In stage V, the SPMTs are disassembled as the gametocyte migrates from the bone marrow to the peripheral blood of the human host ready for transmission to a mosquito (23, 24).

A recent study using cryo-electron tomography revealed that *P. falciparum* gametocytes exhibit a wide range of MT architectures including canonical 13 protofilament MTs, giant 18 protofilament MTs, as well as doublets and triplets with random polarity. Additional SPMTs show a uniform distance to the IMC (25, 26). While the molecular basis of this intimate association between SPMTs and IMC is not understood, the biogenesis and architecture of the IMC during gametocytogenesis have been visualized and found to be constructed from 13 to 15 individual IMC cisternae that are connected at transverse sutures (27–29). These sutures are intervening, proteinaceous regions (28, 30) that might play a role in organizing and stabilizing the cisternal IMC and the underlying SPMTs (29). Microtubule-associated proteins (MAPs), decorating the SPMTs, are also thought to play an important role in these structures. MAPs are expected to define SPMT subpopulations with different properties, to modulate MT stability, mechanical properties, and resistance to depolymerization (9, 25, 31, 32). We recently characterized the *Plasmodium*-specific, SPMT-associated protein *Pf*SPM3 (PF3D7_1327300). Albeit dispensable for asexual blood stage development, knockdown or knockout of *Pf*SPM3 caused severe morphological defects during gametocytogenesis, leading to the formation of round instead of falciform gametocytes and aberrant SPMT organization (21).

Here, we leveraged proximity-based biotinylation utilizing *Pf*SPM3 to characterize the protein network associated with the SPMTs during gametocytogenesis. Furthermore, we mapped the localization of a subset of proxiome-identified proteins and characterized a novel SPMT-interacting factor, offering new insights into the molecular landscape of the gametocyte MT network.

## RESULTS

### Identification of *Pf*SPM3 proxiome by proximity-dependent biotinylation (BioID)

**Fig 1:**
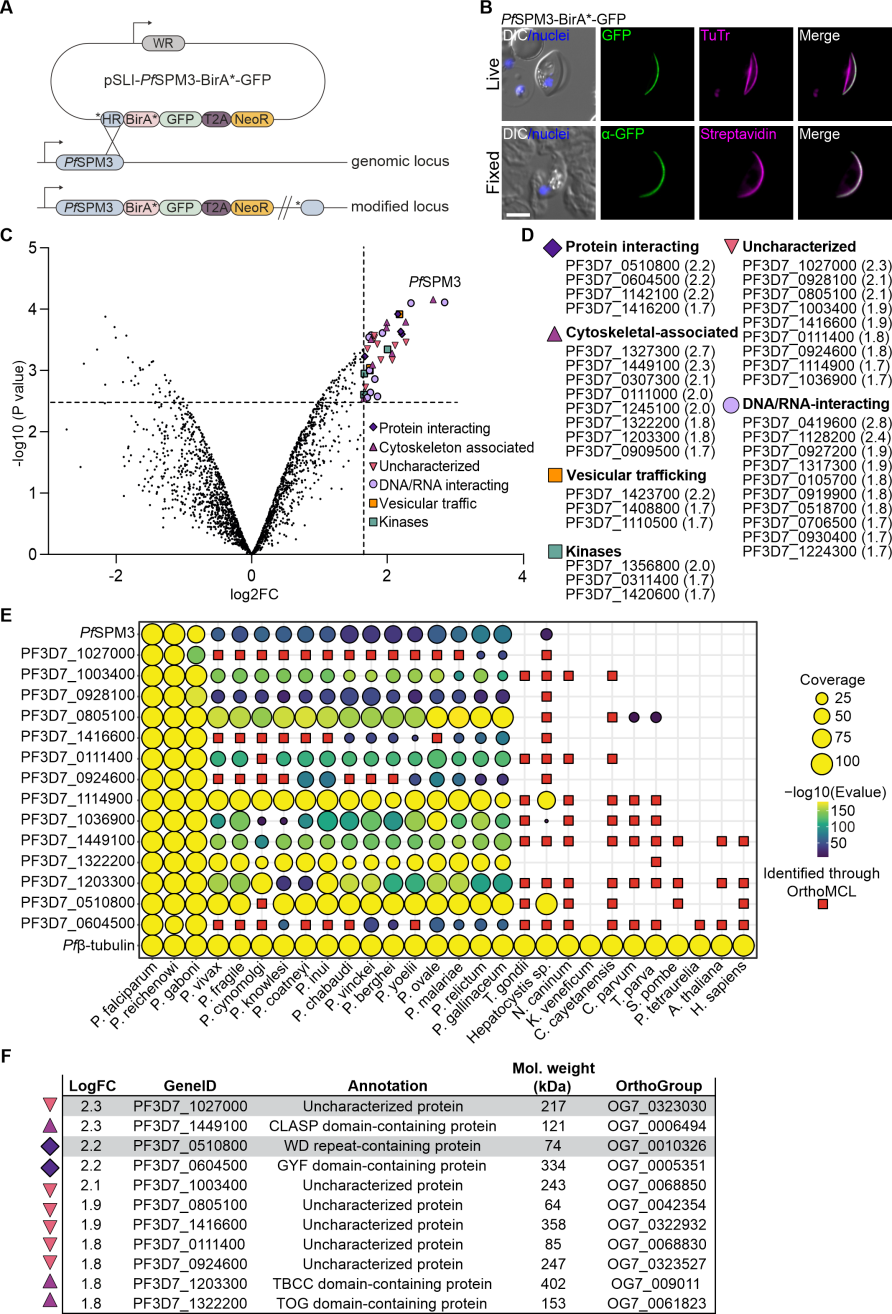
Identification, classification, and conservation of *Pf*SPM3 interacting candidates using BioID. (**A**) Schematic representation of the SLI-based single-crossover homologous recombination approach used to endogenously tag *Pf*SPM3 with a BirA*-GFP cassette at its C-terminus. HR, homology region with an additional stop codon (*); BirA*, biotin ligase; GFP, green fluorescent protein; T2A, skip peptide; NeoR, Neomycin resistance cassette; WR, human dihydrofolate reductase (hDHFR). (**B**) Co-localization of the *Pf*SPM3-BirA*-GFP with MTs. Top row, live-cell fluorescence microscopy images showing *Pf*SPM3-BirA*-GFP (green) and TuTr (magenta). Bottom row: Immunofluorescence microscopy of fixed parasites expressing *Pf*SPM3-BirA*-GFP. *Pf*SPM3-BirA*-GFP (green) was detected using mouse anti-GFP antibodies, and biotinylated proteins were visualized with streptavidin-594 (magenta). Nuclei were stained with Hoechst 33342 (blue). Scale bar, 5 µm. (**C**) Volcano plot from mass spectrometry data depicting the magnitude (log2 FC) and significance (−log10 [*P* value]) of parasite proteins derived from the *Pf*SPM3-BirA*-GFP gametocytes compared to stage-matched parental iGP-3D7 gametocytes. The horizontal dashed line represents the statistical significance threshold corresponding to −log10 (0.0028) = 2.55, and the vertical dashed line shows where the FC is 1.65 [log2 (3.14) = 1.65]. Proteins above this cutoff value are colored according to their functional annotation in PlasmoDB (37) and summarized in (**D**). (**D**) Summary table of proteins in the *Pf*SPM3-associated proxiome that are above the defined cutoff grouped according to predicted molecular functions, including their mean log2 FC values with the biological triplicates. (**E**) Graphical representation of the presence or absence of orthologs for 16 selected proteins from the *Pf*SPM3 proxiome, including *Pf*MSP3 (PF3D7_1327300) and outgroup protein *Pf*β-tubulin (PF3D7_1008700), across 28 species of *Plasmodium* or other eukaryotes was determined using pBLAST. Query coverage indicated by circle size and alignment significance (*E* value) indicated from 0 (dark blue) to ~175 (yellow). Further orthologs were identified using OrthoMCL (red squares). Tubulin beta chain protein (*Pf*β-tubulin) was used as a control (**F**). Tabulated summary of gene annotations and expected protein molecular weight for the 11 proteins identified in the *Pf*SMP3 proxiome that were successfully endogenously tagged. Gray marking highlights the GFP-tagged proteins that show cytosolic localization and were therefore not chosen to be further investigated.

To investigate the protein network associated with the SPMTs during gametocyte development, we performed a BioID proximity labeling assay (33) using *Pf*SPM3 as a bait. We first generated a transgenic cell line by endogenously fusing the C-terminus of *Pf*SPM3 with the BirA* biotin ligase and a GFP tag, using the selection-linked integration (SLI) system (34, 35). The resulting *Pf*SPM3-BirA*-GFP cell line (Fig. 1A and B) was generated on a 3D7-inducible gametocyte producer (iGP) background, which enables robust induction of sexual commitment with a conversion rate of approximately 70% (36).

Expression of *Pf*SPM3-BirA*-GFP and its localization in gametocytes along the tubulin marker Tubulin Tracker (TuTr) was confirmed by live fluorescence microscopy (Fig. 1B, first row). It revealed the previously established co-localization along the SPMTs (21). Biotin addition led to a biotinylation pattern spatially aligned to GFP-tagged *Pf*SPM3, confirming active biotinylation in close proximity to the bait protein (Fig. 1B, second row).

Upon induction of gametocytogenesis (21, 36) in the *Pf*SPM3-BirA*-GFP and parental 3D7-iGP (negative control) cell lines, stage III and IV gametocytes (~8 days post-induction) were supplemented with 0.15 mM biotin for 24 h. Biotinylated proteins from three independent biological replicates were affinity-purified from parasite lysates using streptavidin-coated Sepharose beads and subsequently identified by mass spectrometry. Quantitative analysis comparing peptide enrichment between *Pf*SPM3-BirA*-GFP and the wild-type (WT) 3D7-iGP gametocytes identified 181 proteins with at least twofold enrichment (Table S1) and 49 with at least threefold enrichment (Fig. 1C, colored upper right quartile: cutoff values of log2 fold change [FC] > 1.65). To define high-confidence candidates, proteins with a −log10 (*P* value) lower than that of *Pf*SPM1 (PF3D7_0909500)—another established SPMT-associated protein (21, 38)—were excluded; this left us with 37 proteins that were highly enriched over the control (Fig. 1D).

To refine the *Pf*SPM3-based proxiome, we categorized the >3-fold enriched proteins according to their predicted cellular functions using the integrative database of the malaria parasite PlasmoDB (37). This resulted in a list that comprises four proteins annotated in PlasmoDB according to gene ontology as “protein interacting,” 8 as “cytoskeletal-associated,” 9 as “uncharacterized,” 10 as “DNA/RNA-interacting”, 3 as “vesicular trafficking,” and 3 as “kinases” (Fig. 1D).

To select proteins for further analysis and validation as putative *Pf*SPM3 interacting proteins, we prioritized for this study candidates from the functional categories “protein interacting,” “cytoskeletal-associated,” and “uncharacterized.” Within the “protein interacting” group, PF3D7_1142100 was identified as a chaperone protein (39), and PF3D7_1416200 is annotated as a putative metacaspase-3; both were therefore excluded from further characterization. From the “cytoskeletal-associated” protein group, *Pf*SPM3 (bait) and the following candidates were excluded based on previously reported characterizations: kinesin-8 (PF3D7_0111000 [40]) and kinesin-13 (PF3D7_1245100 [41]), EB1 (PF3D7_0307300 [42]), and *Pf*SPM1 (21). This resulted in a final selection of 14 proteins within the *Pf*SPM3 proxiome for further experimental exploration (Fig. 1E).

### Selected *Pf*SPM3 interacting candidates are mostly *Plasmodium* or Apicomplexa-specific

The phylogenetic relationships of the 14 selected proteins from the *Pf*SPM3 proxiome along with *Pf*SPM3 itself were analyzed using local alignment searches in comparison to a control protein, the tubulin beta chain (*Pf*β-tubulin, PF3D7_1008700) which is well conserved amongst all species (Fig. 1E). Application of these methods revealed several non-parsimonious phylogenetic relationships among *Plasmodium* spp., prompting further ortholog prediction using OrthoMCL (43, 44), which subsequently resolved these discrepancies.

These analyses suggest that all 15 proteins (including *Pf*SPM3) are conserved across *Plasmodium*, and 14 of them are also conserved in the paraphyletic genus *Hepatocystis* (45). Five proteins (*Pf*SPM3, PF3D7_1027000, PF3D7_0928100, PF3D7_1416600, and PF3D7_0924600) appear to be restricted to *Plasmodium*, while an additional five (PF3D7_1003400, PF3D7_0805100, PF3D7_0111400, PF3D7_1114900, and PF3D7_1036900) are exclusive to the phylum Apicomplexa (Fig. 1E). Four proteins (PF3D7_1449100, PF3D7_1203300, PF3D7_0510800, and PF3D7_0604500) have orthologues in distantly related eukaryotes, including plants and humans (Fig. 1E).

### Spatial localization of *Pf*SPM3 proxiome reveals novel MAPs

**Fig 2:**
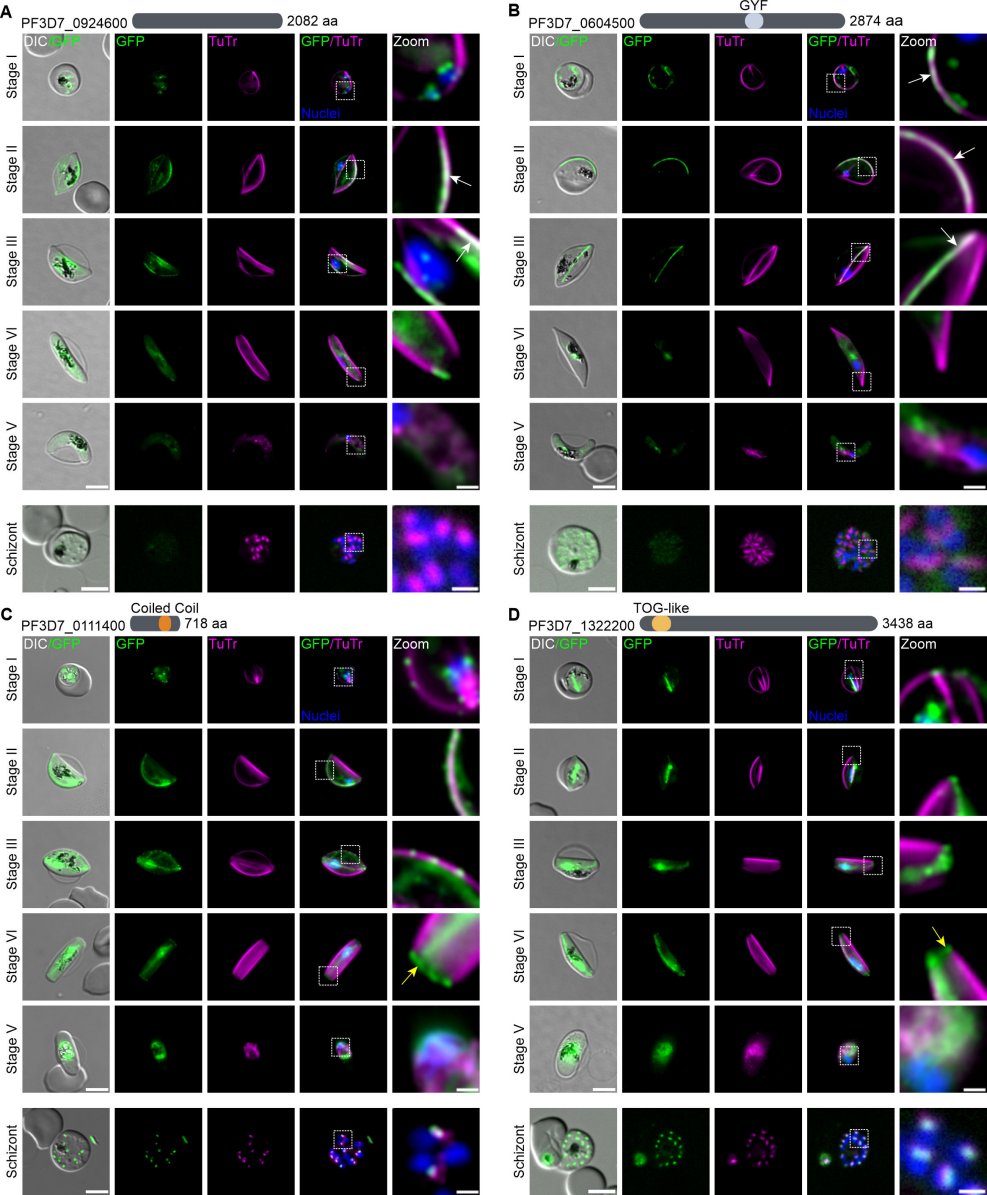
*Pf*SPM3 interacting candidates with microtubular association. (**A–D**) Candidates PF3D7_0924600 (**A**), PF3D7_0604500 (**B**), PF3D7_0111400 (**C**), and PF3D7_1322200 (**D**), showing a schematic representation of their length in aa and predicted domains at the top of each panel, were endogenously tagged with GFP using the SLI system (34, 35). Fluorescence microscopy examination was performed every 2 days starting 72 h after gametocytogenesis induction to capture the co-localization of GFP-tagged proteins (green) with TuTr-labeled MTs (magenta) during gametocyte development (stages I–V). The localization of these candidates was also examined in asexual blood stages (schizont). White arrows indicate co-localization of the candidates PF3D7_0924600 or PF3D7_0604500 with TuTr. Yellow arrows indicate a concentration of the candidates PF3D7_0111400 and PF3D7_1322200 at the distal end of stage IV gametocytes. Zoom, enlargement of the image section. Nuclei were stained with Hoechst 33342 (blue). DIC, differential interference contrast. Scale bar, 5 and 1 µm for zoomed sections.

To investigate the subcellular localization of the 14 selected proteins, we targeted the corresponding genes using SLI-based gene editing to generate C-terminal GFP-tagged proteins (34) (Fig. S1A). All parasite lines were generated in NF54/iGP parasites (36). Gene-specific integration via homologous recombination mediated by the SLI-based constructs was confirmed by diagnostic PCR (Fig. S1A and B). Despite multiple attempts, we were unable to generate transgenic parasite lines for three of the target proteins (PF3D7_0928100, PF3D7_1114900, and PF3D7_1036900), with all of these refractory candidates belonging to the group of “uncharacterized” proteins. Due to the inability to tag these proteins, they were excluded from further analysis. Therefore, we proceeded with downstream analysis for the remaining 11 successfully tagged proteins (Fig. 1F). After induction of gametocytogenesis, expression of these tagged proteins was confirmed by fluorescence microscopy with additional western blot analysis for those candidates with a mass below 250 kDa (PF3D7_0805100, PF3D7_1203300, and PF3D7_1449100) (Fig. S1C). Of note, although the PF3D7_0111400-GFP protein is clearly expressed and associated with the SPMTs in asexual (schizont) stage and gametocytes (Fig. 2C), we were not able to detect this candidate on western blots. This might be due to the high degree of intrinsically disordered regions of this protein (24.4% compared to 9.2% in PF3D7_0805100, 12.5% in PF3D7_1203300, and 14.7% in PF3D7_1449100, as predicted by the SEG algorithm [46]) and its unusual high Asparagine content (22% compared to 14% for PF3D7_0805100, 16% for PF3D7_1203300 and 17% for PF3D7_1449100).

Two candidates (PF3D7_0510800 and PF3D7_1027000) exhibited low levels of expression of the tagged protein with an ambiguous distribution pattern which led to their exclusion from further validation.

**Fig 3:**
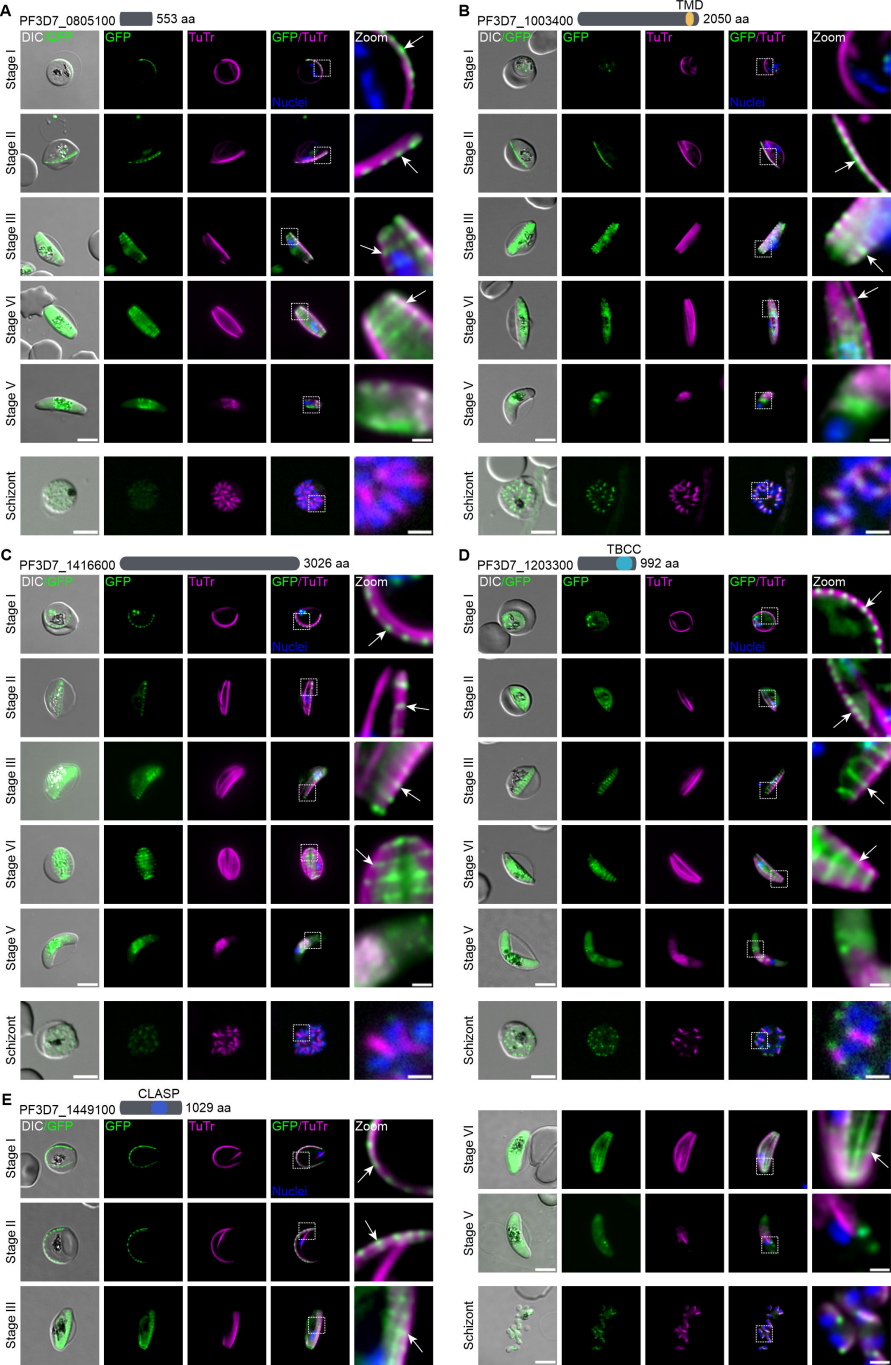
*Pf*SPM3 interacting candidates with IMC suture-like distribution. (**A–E**) Candidates PF3D7_0805100 (**A**), PF3D7_1003400 (**B**), PF3D7_1416600 (**C**), PF3D7_1203300 (**D**), and PF3D7_1449100 (**E**), showing a schematic representation of their length in aa and predicted domains at the top of each panel, were endogenously tagged with GFP using the SLI system (34, 35). Fluorescence microscopy examination was performed every 2 days starting 72 h after gametocytogenesis induction to capture the co-localization of GFP-tagged proteins (green) with TuTr-labeled MTs (magenta) during gametocytogenesis (stages I–V). The localization of these candidates was also examined in asexual blood stages (schizont). White arrows show a suture-like pattern for each candidate. Zoom, enlargement of the image section. Nuclei were stained with Hoechst 33342 (blue). DIC, differential interference contrast. Scale bar, 5 and 1 µm for zoomed sections.

Imaging of the resulting nine transgenic parasite lines demonstrated a dynamic localization pattern of these candidates during gametocyte maturation from stages I to V (Fig. 2 and 3). At each time point, MTs were stained with TuTr to visualize the co-localization of the GFP-tagged proteins. Four of the target proteins showed patterns of association with the MT networks: PF3D7_0924600 (conserved unknown protein) reveals a *Pf*SPM3-like pattern and localizes to the SPMT network until disappearing during transition between stages IV and V (Fig. 2A). PF3D7_0604500 (glycine-tyrosine-phenylalanine domain [GYF]-containing protein) appears to localize prominently to some, but not all, SPMTs. It disappears earlier during gametocytogenesis, from stage IV onwards (Fig. 2B). The two other proteins—PF3D7_0111400 (harboring a coiled-coil domain) and PF3D7_1322200 (containing a tumor overexpressed gene [TOG] domain)—appear to be primarily associated with intranuclear MTs but also show some SPMT association, as well as a concentration of the tagged proteins at the distal end of stage IV gametocytes (Fig. 2C and D).

Notably, five tagged proteins (PF3D7_0805100, PF3D7_1003400, PF3D7_1416600, PF3D7_1203300, and PF3D7_1449100) display a distinct, yet spatially associated localization relative to the SPMTs (Fig. 3). They are concentrated in defined SPMT subregions, which subsequently resolve in a transversal pattern as gametocytes develop from stages III to IV (Fig. 3A through E). This pattern is reminiscent of the distribution of IMC suture proteins (28, 30) as they do not appear to be equally distributed within IMC sutures but appear to be concentrated at the intersection with the SPMT network.

RNA-seq analysis (data available on PlasmoDB [47]) was conducted in order to differentiate the sex ratio of these nine selected proteins. Using PF3D7_1250100 (osmiophilic body protein *Pf*G377) and PF3D7_1325200 (putative lactate dehydrogenase, *Pf*LDH2) as markers for female (48) and male (49) gametocytogenesis, respectively, the analysis revealed that four proteins (PF3D7_0924600, PF3D7_0805100, PF3D7_1416600, and PF3D7_1003400) show an elevated expression level in female gametocytes, whereas the other five (PF3D7_1449100, PF3D7_1322200, PF3D7_1203300, PF3D7_0604500, and PF3D7_0111400) were found to be preferentially expressed in male gametocytes (Fig. S2A and B).

The expression and localization of these nine proteins was also investigated in asexual (schizont) stages of the parasites. Five of them (PF3D7_0111400 and PF3D7_1322200 in Fig. 2C and D; PF3D7_1003400, PF3D7_1203300, and PF3D7_1449100 in Fig. 3B, D and E) were detectable by fluorescence microscopy and revealed the expected association with the SPMTs. Notably, PF3D7_1203300 appears to be concentrated at the tips of the SPMTs. The remaining four tagged proteins (PF3D7_0924600, PF3D7_0604500, PF3D7_0805100, and PF3D7_1416600) exhibited reduced expression levels or were undetectable by fluorescence microscopy during the schizont stage (Fig. 2 and 3).

**Fig 4:**
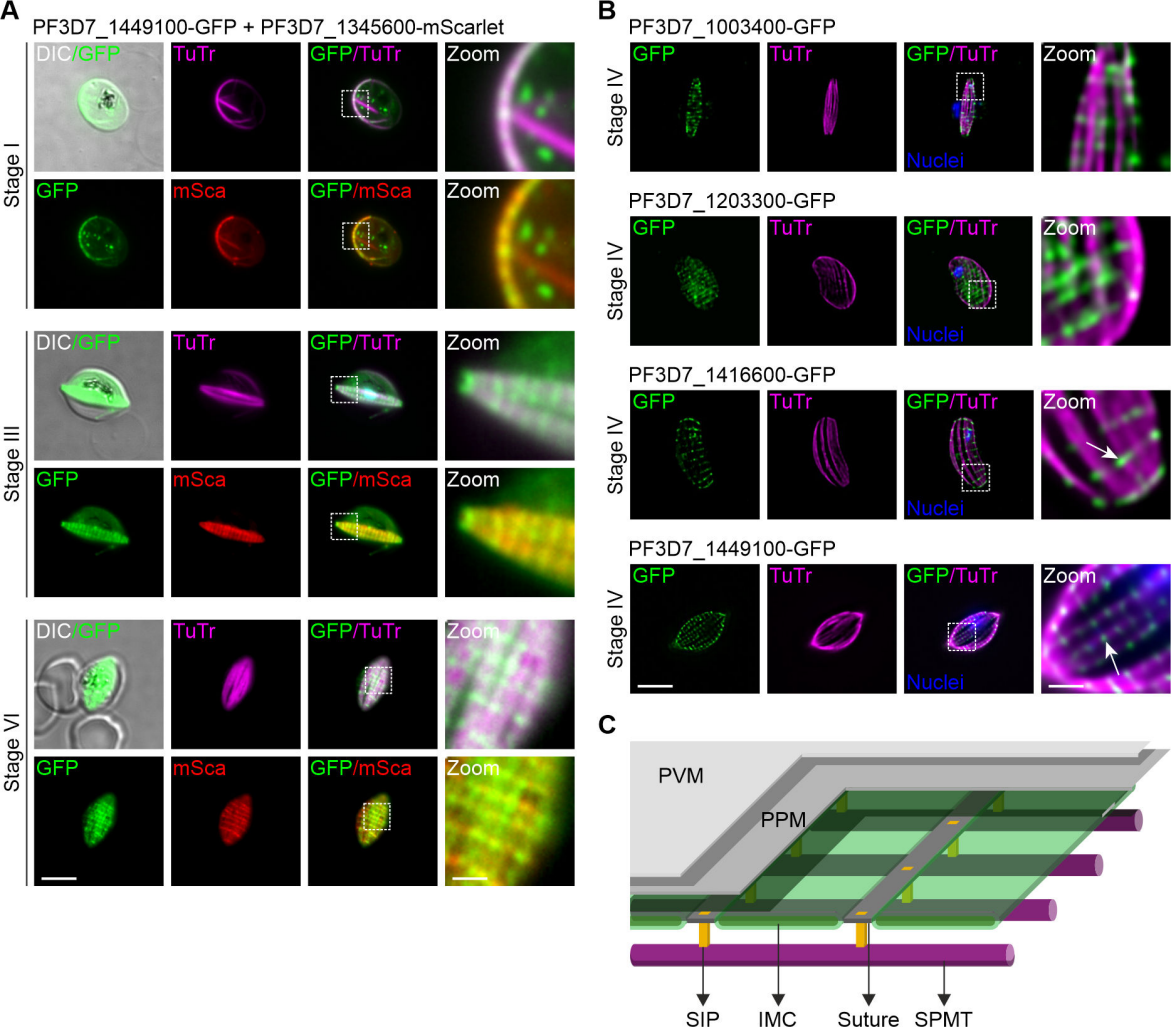
Co-localization of suture-like candidates with the subpellicular microtubule (SPMT) network. (**A**) Candidate PF3D7_1449100 (green) was colocalized with TuTr (magenta) and the IMC suture marker PF3D7_1345600 (red) at gametocyte stages I, III, and IV. (**B**) Fluorescence microscopy data based computational clearing (Thunder software, Leica) of images derived from PF3D7_1003400-GFP (first row), PF3D7_1203300-GFP (second row), PF3D7_1416600-GFP (third row), and PF3D7_1449100-GFP (last row) reveals discrete concentration of the suture-like proteins (see arrows, green) in close proximity to the SPMTs visualized by TuTr (magenta). Nuclei were stained with Hoechst 33342 (blue). Zoom, enlargement of the image section. Scale bar, 5 and 1 µm for zoomed sections. (**C**) Schematic model illustrating a potential organization of the IMC and SPMTs at the pellicle of the gametocytes: SPMTs are anchored to the IMC through suture interacting proteins (SIPs) that could ensure a spatial link and even spacing between the IMC and SPMTs. PPM, parasite plasma membrane; PVM, parasitophorous vacuole membrane.

In order to investigate the IMC suture-like distribution in gametocytes, we endogenously tagged the *bona fide* IMC suture marker PF3D7_1345600 (28) with mScarlet (Fig. 4A) in the PF3D7_1449100-GFP expressing parasite line using the SLI2 vector system (50). Live-cell fluorescence imaging revealed that the distribution of the PF3D7_1449100 is indeed restricted to these specialized areas of the IMC, with a similar localization to that of the suture marker protein PF3D7_1345600, but appears to be confined to foci within the sutures (Fig. 4A). To explore this distribution in greater detail, we applied image processing using Leica Thunder software to the widefield fluorescence microscopy data for each of the GFP-tagged proteins seen to have a suture-like distribution (PF3D7_1003400, PF3D7_1203300, PF3D7_1416600, and PF3D7_1449100). This approach revealed some discrete transversal GFP foci that are closely associated with the SPMT network (most pronounced in PF3D7_1449100-GFP; Fig. 4B), with an overall similar but not identical spatial distribution between the individual proteins (Fig. 4B), potentially representing a molecular nexus of the SPMT with the IMC architecture (Fig. 4C).

### Deletion of the suture-like protein PF3D7_1003400 is linked to a defect in gametocytogenesis

**Fig 5:**
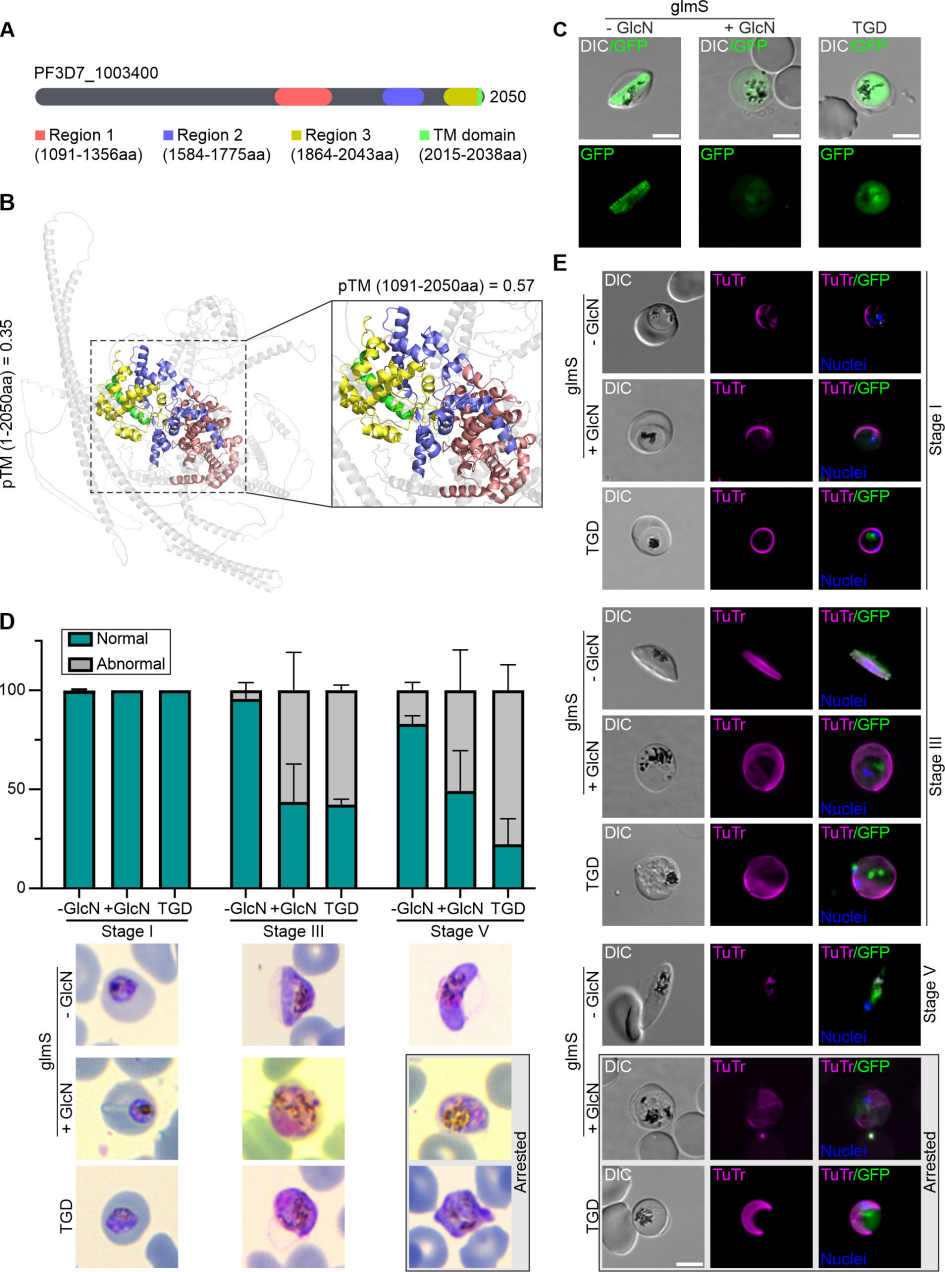
Knockdown or truncation of the suture-like protein PF3D7_1003400 leads to an arrest of gametocytogenesis and loss of falciform shape. (**A**) Schematic representation of the protein PF3D7_1003400. The identification of conserved regions and a transmembrane (TM) domain was determined by sequence alignment of PF3D7_1003400 and its orthologs (see Fig. S3). Regions 1, 2, and 3 are shown in red, blue, and yellow, respectively. TM domain is shown in green. Non-conserved regions are shown in gray. (**B**) Structural modeling of PF3D7_1003400 is based on Alphafold3 (53). Conserved regions and TM domain of PF3D7_1003400 are shown as in (**A**). Zoomed area of the conserved regions and TM is shown next to the model. pTM values for the whole (1–2,050 aa) and the conserved section (1,091–2,050 aa, which include regions and TM domain) of PF3D7_1003400 are 0.35 and 0.57, respectively. (**C**) Live-cell microscopy images of gametocytes expressing the full-length (−GlcN) or conditional knockdown (+GlcN) of PF3D7_1003400-GFP-glmS, and the truncated version (TGD) of PF3D7_1003400-GFP. (**D**) Quantification of gametocyte morphology of control (−GlcN) and knockdown (+GlcN) PF3D7_1003400-GFP-glmS, and truncated PF3D7_1003400 (TGD) gametocytes at stages I, III, and V. Bar graph shows the percentage of gametocytes with normal (falciform shape from stage III) and abnormal (non-falciform shape) morphology. Representative Giemsa-stained images of gametocytes at the respective stages are shown below. (**E**) Live-cell fluorescence microscopy examination of control (−GlcN) and knockdown (+GlcN) PF3D7_1003400-GFP-glmS, and truncated PF3D7_1003400 (TGD) gametocytes at stages I, III, and V. MTs were labeled with TuTr (magenta). Gray boxes in (**D and E**) show an arrested gametocyte phenotype in conditional knockdown and TGD parasites at stage V. Nuclei were stained with Hoechst 33342 (blue). DIC, differential interference contrast. Scale bar, 5 µm.

To further investigate the functional role of suture-like proteins forming discrete foci at the SPMTs during gametocytogenesis, we selected the uncharacterized protein PF3D7_1003400. It contains 2,050 amino acids (aa), and orthologs are present in different *Plasmodium* species and other apicomplexans (Fig. 1E). A detailed analysis of the sequence alignment of PF3D7_1003400 and its orthologs revealed the presence of three conserved regions toward the C-terminus of the protein (Fig. 5A and B; Fig. S3). PF3D7_1003400 and its orthologs end with a hydrophobic region with a predicted alpha helical configuration (2,015–2,038 aa). In the case of PF3D7_1003400 (and most of its orthologs), this part is predicted by TMHMM (51) as a potential transmembrane (TM) domain (Fig. 5A and B; Fig. S3). Despite the evident conservation of these regions, HHpred search (52) did not show any predicted domain functions.

To gain some insight into the function of PF3D7_1003400, we employed two different reverse genetic approaches: a conditional knockdown and targeted gene disruption (TGD) of PF3D7_1003400. The first approach inserted the glmS ribozyme sequence upstream of the 3′ translated region of the Neomycin resistance (NeoR) cassette (54), thereby producing the endogenously expressed PF3D7_1003400-GFP-T2A-NeoR-glmS (Fig. S1A). The conditional knockdown of PF3D7_1003400 could be induced upon the addition of 2.5 mM glucosamine (GlcN), resulting in the degradation of mRNA and reduced expression of the GFP signal (Fig. 5C). The second approach used the SLI-TGD system (34) that led to the expression of a truncated GFP-tagged remnant of PF3D7_1003400 comprising 211 out of 2,050 aa. Successful gene disruption of PF3D7_1003400 was confirmed by PCR and western blot (Fig. S1D) and showed no detrimental effect on blood stage parasite growth despite localizing to the SPMTs in blood stages, indicating that this gene is dispensable for asexual replication of the parasite *in vitro*.

Subsequently, we investigated the potential impact of the gene modifications on localization and gametocytogenesis. The GFP-tagged deletion mutant, which displayed none of the conserved regions including the predicted TM domain, was no longer associated with SPMTs and instead localized to the cytosol (Fig. 5C). Importantly, the loss of PF3D7_1003400 by either the glmS or TGD systems resulted in abnormalities in gametocyte morphology (Fig. 5D and E). Gametocytogenesis appears to be arrested somewhere during stage III progression and resulted in accumulation of vesicles (Fig. 5D and E). While early-stage gametocyte development appeared largely unaffected (stage I: 99.6% normal in WT compared to 100% in glmS and 100% in TGD), an abnormal (defined as aberrant non-falciform phenotype) morphology was observed from stage III onwards, with 56.3% and 57.7% of the glmS and TGD gametocytes, respectively, in contrast to the typical morphology shown in most of the WT gametocytes (95.7%) (Fig. 5D and E). At stage V, 50.9% and 77.7% of the glmS and TGD gametocytes exhibited abnormal morphology, respectively, whereas 83.1% of WT gametocytes were falciform (Fig. 5D and E). Interestingly, TuTr was still associated with the MT network in glmS and TGD gametocytes (Fig. 5E).

## DISCUSSION

*P. falciparum* gametocytes—the pre-sexual stages of the parasite that enable transmission from humans to the mosquitos—develop into elongated and rigid cells as they mature within red blood cells in the bone marrow. These maturing gametocytes then re-emerge in the peripheral blood as mature “falciform” cells with a depolymerized and collapsed SPMT network (29). To date, the molecular mechanisms by which the parasite organizes, maintains, and disassembles this unique variety of SPMTs remain unclear. Additionally, the nature of the connections between the SPMT network and the IMC inner leaflet, maintained at an approximate distance of 20 nm, is still not fully understood (11). The SPMT connection to the IMC is thought to be mediated by specific proteins such as *Pf*SPM3, with loss of *Pf*SPM3 leading to a collapse of the SPMT network and changes in *P. falciparum* gametocyte shape (21). Here, using BioID, we identified proteins in close spatial association with *P. falciparum* SPM3. We demonstrate the partial co-localization of several of these proteins with the SPMTs and specialized structures known as sutures and show that loss of one such protein recapitulates the gametocyte rounding phenotype observed in *Pf*SPM3-deficient parasites (21).

Functional classification of BioID-identified putative *Pf*SPM3 interacting partners revealed an enrichment of cytoskeletal proteins, vesicle trafficking proteins, and proteins that interact with other proteins across the high confidence putative interacting partners (Fig. 1C and D). This suggests that *Pf*SPM3 operates within a complex protein network interfacing with the SPMTs. Phylogenetic analysis of the top candidates revealed a mosaic of conservation with 10 out of 14 proteins specific to Apicomplexa. Several proteins appear to be well conserved within the *Laverania* clade but diverge in *Plasmodium* species more distantly related to *P. falciparum* (e.g., PF3D7_1027000 or PF3D7_1416600). This could imply that these proteins have evolved certain cytoskeletal adaptations specific to the falciform gametocytogenesis that are a hallmark of the *Laverania*.

Using a C-terminal GFP tag, we determined the localization of 9 proteins of the *Pf*SPM3 proxiome that show different patterns of MT association ranging from mainly SPMT associations (PF3D7_0604500 and PF3D7_0111400) to MT/nuclei and SPMT-end associated (PF3D7_1322200). In addition, we have increased the number of proteins known to be associated with transversal structures reminiscent of IMC sutures from two (PF3D7_1345600 and DHHC1) (28, 30) to seven with the inclusion of PF3D7_0805100, PF3D7_1003400, PF3D7_1416600, PF3D7_1203300, and PF3D7_1449100. These five proteins show a distinct spatial pattern, forming transversal suture-like structures with accumulations at the intersection with the SPMTs, suggesting a role in supporting the alignment of MTs (28, 29, 55). Here, we provide functional evidence that one IMC suture-related protein, PF3D7_1003400, is involved in the morphological integrity of the *P. falciparum* gametocyte as conditional knockdown and targeted disruption of this protein led to rounding up of *P. falciparum* gametocytes. This phenotype correlated with a breakdown in the spatial organization of the SPMTs. This suggests that PF3D7_1003400 is important in maintaining the structural integrity of the pellicle by ensuring the precise alignment and anchoring of SPMTs at the sutures. Interestingly, this 243 kDa protein displays a C-terminal hydrophobic region that is predicted to be a TM domain. A schematic structural model depicted in Fig. 4C highlights the potential central role of these SPMT/suture-interacting proteins in anchoring the SPMTs to the IMC sutures, forming a stable cytoskeletal framework for establishing the falciform morphology of *P. falciparum* gametocytes.

MAPs have been well characterized in the related apicomplexan parasite *Toxoplasma gondii*. In tachyzoite stages, a stage whose SPMT distribution resembles partially that of gametocytes, MAPs coat the entire length of the SPMT or exhibit distinct patterns along the polymers (38, 56, 57). Anchoring of MTs along the IMC via the alveolar protein GAPM1a has been shown in *T. gondii*, playing a role in MT stability and organization (58). Similar findings have been described for the tethered protein IMC and SPMT protein 1 (IMT1), that forms a bridge from the SPMTs to the IMC (59). It has been recently reported that MT nucleation—driven by γ-tubulin and γ-tubulin complex proteins 4, 5, and 6 (GCP4/5/6), which are components of the γ-tubulin ring complex—occurs at distinct sites on the apical polar ring, suggesting a key role in organizing the daughter scaffold formation through the nascent SPMT rafts (60). Stage and species-specific phenotypes have been demonstrated by inactivation of MAPs in different *Plasmodium* species (21, 61).

Some of the *Pf*SPM3 interacting proteins display well-established domains involved in MT dynamics. Cytoplasmic linker-associated protein (CLASP) domain-containing proteins, such as PF3D7_1449100, are typically key regulators in MT organization and dynamics. They belong to plus-end tracking proteins (+TIPs), which are essential regulators of MT dynamics and associate with the distal ends of MTs to stabilize specific MT subsets (62). In this study, we demonstrate that the TOG domain-containing protein PF3D7_1322200 also shows a clear association with the SPMT ends (Fig. 3D). TOG domain-containing proteins, such as PF3D7_1322200, are best known for their ability to bind to tubulin heterodimers (63), opening up a potential avenue for further exploring the molecular function of PF3D7_1322200. The tubulin binding cofactor C (TBCC, associated with folding of tubulins) domain-containing protein PF3D7_1203300 also localized with an IMC suture-like pattern, with foci at the intersection with the SPMTs (Fig. 5). These structural predictions provide potential insights about how these proteins may function and interact with the SPMT network, but future work will be needed to fully elucidate the role of these domains in the localization and function of these proteins.

Taken together, the proteins characterized in this study, as well as some of the remaining interacting partners within the *Pf*SPM3 proxiome, could potentially play a role in linking the SPMT with the IMC and IMC sutures. This underlying and not yet fully characterized protein network includes essential proteins required for normal gametocyte development and likely successful infection of the mosquito vector. We demonstrate that the intimate and spatially coordinated interplay of the SPMTs and IMC is a requirement of gametocyte morphogenesis. The concentration of malaria/apicomplexan-specific proteins we identified to be involved in this network highlights the potential of targeting key and potentially unique protein-protein interactions with inhibitors to disrupt gametocyte development and malaria transmission.

## MATERIALS AND METHODS

### *P. falciparum* culture

Asexual blood stage 3D7-iGP1 (for BioID) and NF54-iGP2 *P. falciparum* (36) were cultured in human B+ erythrocytes (obtained from the Institute of Transfusion Medicine, Universitätsklinikum Eppendorf, Hamburg) at 5% hematocrit in RPMI-1640 complete medium supplemented with 0.5% Albumax II, 25 mM HEPES. Cultures were maintained at 37°C in an atmosphere of 1% O_2_, 5% CO_2_, and 94% N_2_. Culture synchronization was achieved using 5% d-sorbitol (64) or 60% Percoll (65).

Induction of gametocytogenesis in the SPM3-BirA*-GFP-3D7/iGP1 parasite line was done as previously described (36, 41). Briefly, sorbitol-synchronized rings (2–5% parasitemia) were induced by addition of Shield-1 to a final concentration of 1.25 µM to the culture medium with the aim to stabilize the GDV1-GFP-DD expression, with media and Shield-1 changed daily. After 72 h on Shield-1, cultures were fed daily with RPMI containing 0.25% Albumax and 5% human serum. To eliminate asexual parasites, 50 mM N-acetyl-d-glucosamine (GlcNAc) was added for 5 days starting from day 3. Cultures of NF54/iGP2-based parasites were cultured in the presence of 2.5 mM GlcN to prevent overexpression of the gametocyte commitment factor GDV1 (36), and medium was supplemented with 2 mM choline.

### Plasmid construction

For the endogenous tagging of *Pf*SPM3 with BirA*-GFP, a homology region of the last 1,021 bp before the stop codon was amplified from 3D7 gDNA and cloned into the plasmid pSLI-Phil1-BirA*-GFP (66) by using the NotI and AvrII (NEB) restriction sites replacing the Phil1 homology region and creating the transfection vector pSLI-*Pf*SPM3-BirA*-GFP. For endogenous tagging of the selected candidates, a homology region of approximately 1,000 bp upstream of the stop codon of the following genes: PF3D7_1114900, PF3D7_1322200, PF3D7_1203300, PF3D7_1027000, PF3D7_0928100, PF3D7_0924600, PF3D7_1416600, PF3D7_0604500, PF3D7_0805100, PF3D7_0111400, PF3D7_0510800, and PF3D7_1036900 were cloned into pSLI-VPS11-2xFKBP-GFP-2xFKBP_1xNLS-FRB-T2A-hDHFR (67, 68) by using the restriction enzymes NotI and AvrIII (NEB) to replace the VPS11 sequence.

The homology region of PF3D7_1003400 was inserted in pSLI-GFP-glmS (35) using the NotI and MluI (NEB) restriction sites.

For SLI-TGD (34) of PF3D7_1003400, the 613 bps downstream of the gene start (ATG) codon was amplified from 3D7 gDNA by PCR and cloned into the pSLI-TGD plasmid using NotI and MluI (NEB) restriction enzymes. For double endogenous tagging of a suture marker protein, a homology region of approximately 1,000 bp upstream of the stop codon of the gene PF3D7_1345600 was cloned into pSLI2 (50) using NotI and AvrII to generate pSLI2-PF3D7_1345600-mScarlet. For primer sequences, see Table S2.

### Parasite transfection

For transfection of constructs, Percoll (GE Healthcare)-enriched, synchronized mature schizonts were electroporated with 50 µg of plasmid DNA using a Lonza Nucleofector II device (69). Transfectants were selected in medium supplemented with 3 nM WR99210 (Jacobus Pharmaceuticals). For the generation of stable integrant cell lines, parasites containing the episomal plasmids selected with WR99210 were grown with 400 µg/mL Neomycin/G418 (Sigma) to select for transgenic parasites carrying the desired genomic modification as described previously (34). After the second round of selection, genomic DNA was extracted from parasite pellets (Qiagen QIAamp DNA Mini Kit). PCR was performed to confirm the correct integration of target genes using primers designed to amplify across the integration site. For primer sequences, see Table S2.

### BioID and mass spectrometry analysis

The protocol was adapted from previously published BioID approaches (70, 71) and mass spectrometry analysis was performed as described previously (71). Briefly, 100 mL culture of *Pf*SPM3-BirA*-GFP and 3D7-iGP1 was synchronized with sorbitol and ring parasitemia was adjusted to 2–5%. Gametocytogenesis was induced by the addition of Shield-1 for 72 h to the culture medium as described above to stabilize GDV1-GFP-DD expression. Asexual stages were eliminated by treatment with GlcNAc medium as described above. At day 8 after induction, proper development was confirmed microscopically and stage III gametocytes were cultured for 24 h in RPMI containing 0.25% Albumax/5% human serum supplemented with 150 µM biotin (Sigma-Aldrich). Three inductions in consecutive weeks were performed, each as an independent biological replicate. After incubation with biotin, samples were collected for microscopy and gametocyte stages were purified in a 60% Percoll gradient, washed two times with DPBS, and resuspended in lysis buffer (50 mM Tris-HCl pH 7.5, 500 mM NaCl, 1 mM DTT, 1 mM PMSF, 2× protease inhibitor cocktail [PIC], and 1% Triton-X-100) and frozen at −80°C. After three freeze-thaw cycles, lysates were centrifuged at 25,000 × *g* for 60 min at 4°C, and the supernatant was stored at −80°C. For purification of biotinylated proteins, 50 µL streptavidin Sepharose (GE Healthcare) was added to the lysate and incubated overnight with end-over-end rotation at 4°C. The beads were washed two times in lysis buffer, once in dH_2_O, two times in Tris-HCl (pH 8.5) and three times in 100 mM triethylammonium bicarbonate buffer (TEAB) pH 7.5 (Sigma-Aldrich). The washed beads were resuspended in 200 µL 50 mM ammonium bicarbonate (pH 8.3) containing 1 µg of trypsin (Roche) and incubated shaking at 650 rpm for 16 h at 37°C, followed by a second digest with 0.5 µg trypsin for 2 h. Beads and supernatants were transferred onto a spin column (Pierce Spin Columns with Snap Cap, Thermo Scientific) placed in a low binding tube (Low Protein Binding Microcentrifuge tubes, Thermo Scientific) and centrifuged at 2,000 rpm at RT for 5 min. Two elutions with 150 µL 50 mM TEAB pH 8.5 were performed. Eluates were stored at −80°C and further dried using a SpeedVac (Thermo Fisher Scientific) and stored at −20°C. Dried peptides were sent to the Proteomics Core Facility at EMBL Heidelberg and processed as described (71) using a QExactive plus (Thermo) mass spectrometer. The mass spectrometry proteomic data have been deposited to the ProteomeXchange Consortium via the PRIDE (72) partner repository with the data set identifier PXD060994. Full data are also available in Table S1.

### Live-cell microscopy

For staining of nuclei, parasites were incubated with 0.45 µg/mL Hoechst 33342 in culture medium for 30 min at 37°C. Images were acquired on a Leica DM 6B fluorescence microscope, equipped with a Leica DFC9000 GT camera and a Leica Plan Apochromat 100 Å~/1.4 oil objective. MTs were visualized by incubation of parasites in medium containing 1:1,000 TubulinTracker deep red (Thermo Fisher Scientific; dissolved in dimethyl sulfoxide [DMSO]), which labels polymerized tubulin, for 15 min at 37°C prior to imaging, as previously described (73).

### Immunofluorescence assay (IFA)

Stage III gametocytes were fixed in 4% paraformaldehyde and 0.0075% glutaraldehyde for 30 min at RT. Samples were permeabilized with 0.1% Triton X-100 and blocked with 3% bovine serum albumin in phosphate-buffered saline (PBS). IFAs were conducted using primary antibodies specific to GFP (dilution 1:500, mouse, Roche) and secondary goat anti-mouse antibodies coupled to Alexa Fluor 488 (dilution 1:2,000, Invitrogen). For the detection of biotinylated proteins, streptavidin conjugated to Alexa Fluor-594 dyes (Thermo Fisher Scientific) was used in a 1:4,000 dilution. Images were taken with a Leica DMi-8 inverted widefield microscope equipped with a DFC9000 GTC sCMOS camera (Leica, Germany) and a 100×/1.4 NA oil objective.

### Image processing

Image processing for both live-cell and fixed-cell microscopy was performed using *FIJI* (74) and representative images were adjusted for brightness and contrast. For selected micrographs, resolution was enhanced utilizing the THUNDER algorithm (Leica, Germany). Computational clearing was applied with small-volume settings calibrated to a 2,000 nm scale and 60% clearance for the 488 and 658 nm channels, ensuring improved clarity and detail.

### Western blot analysis

For immunoblots, schizont-infected erythrocytes (4–8% parasitemia) were lysed with 0.03% saponin in 1× TBS and washed repeatedly in 1X TBS. The released parasites were then lysed with 4% SDS, 0.5% Triton X-100, and 0.5X PBS containing PIC (Roche). Lysates were centrifuged at maximum speed, and the supernatants were resuspended in 4X SDS sample buffer and boiled at 95°C for 5 minutes. Proteins were separated using 8% polyacrylamide or 4–12% precast (BioRad) gels and transferred to nitrocellulose membranes (GE Healthcare) using a tank blot device (Bio-Rad). The membranes were blocked with 5% milk in TBS containing 0.01% Tween-20 (TBST) for 2 h at RT, followed by the incubation with mouse anti-GFP (1:1,000, Invitrogen), mouse anti-FKBP (1:300, Santa Cruz), or 1:2,000 rabbit anti-aldolase (75) primary antibodies in 2% milk in TBST at 4°C overnight. After three washes with TBST, the membranes were incubated with HRP-conjugated anti-mouse (1:3,000) or anti-rabbit (1:2,500) secondary antibodies in 2% milk in TBST, or alternatively, IRDye 800CW goat anti-mouse (1:20,000, LICORbio) secondary antibody in 3% BSA in TBST, for 1 h at RT. For HRP conjugates, chemiluminescent signals were detected with Clarity Western ECL substrate (Bio-Rad) and recorded with a ChemiDoc XRS imaging system (Bio-Rad); whereas for IRDye conjugates, fluorescent signals were visualized using Odyssey Fc Imager by LICOR Biosciences.

### RNA-sequencing expression analysis

The RNA-sequencing data of *P. falciparum* 3D7 gametocyte stages (47) were obtained from PlasmoDB (v.68). Transcript abundance (in transcripts per million, TPM) was retrieved for the genes of interest in order to assess their sense expression levels. Expression values were compared between male and female gametocytes. PF3D7_1250100 (osmiophilic body protein G377, *Pf*G377) and PF3D7_1325200 (putative lactate dehydrogenase, *Pf*LDH2) were used as markers for female and male gametocytogenesis, respectively. Graphs were performed in R (version 4.4.3) using tidyverse packages.

### Phylogenetic analysis of the *Pf*SPM3 proxiome

Protein sequences of ortholog genes in non-*P*. *falciparum* species were identified by using protein-to-protein BLAST search (blastp). Candidate genes analyzed included PF3D7_0111400, PF3D7_0510800, PF3D7_0604500, PF3D7_0805100, PF3D7_0924600, PF3D7_1003400, PF3D7_1027000, PF3D7_1203300, PF3D7_1322200, PF3D7_1416600, and PF3D7_1449100. The search was performed against the NCBI non-redundant (nr) protein database and excluding “*P. falciparum*” as a species. Coverage and *E* values were extracted for significant hits, and representative species were selected for visualization. Data processing and visualization were performed by R. Sequence coverage was used to determine bubble size and −log(10) of the *E* value was used to decide on color intensity. *E* values equal to 0 were set as the maximum color. R was also used to create the plot itself with genes plotted in the *x*-axis and species on the *y*-axis. Additional orthologs were inferred using OrthoMCL (version 7, Release 7.0 [43, 44]) but were not assigned coverage or E-values.

### Sequence analysis

The identification of conserved regions in the suture-like protein PF3D7_1003400 was performed by sequence alignment of this protein and its orthologs in different *Plasmodium* species and other apicomplexan parasites, using Clustal Omega (76). The potential TM domain or hydrophobic region in PF3D7_1003400 was predicted by TMHMM (51). The representation of these alignment sequences, consensus, and conservation was visualized using Jalview software (77). The structural analysis of PF3D7_1003400 was predicted using AlphaFold3 (53) and the identification of these conserved regions and TM domain was performed using PyMOL.

## Data Availability

The full proteomic data set has been deposited to the ProteomeXchange Consortium via the PRIDE partner repository with the data set identifier PXD060994.

## SUPPLEMENTAL MATERIAL

Fig. S1 mbio.03407-25-s0001.tif Additional data for the selected proteins identified in the PfSPM3 BioID.

Fig. S2 mbio.03407-25-s0002.tif RNA seq expression of candidates in female and male gametocytes.

Fig. S3 mbio.03407-25-s0003.tif Schematic representation of the suture-like protein PF3D7_1003400 and sequence alignment with different orthologs.

Legends mbio.03407-25-s0004.pdf Legends for supplemental files.

Supplemental Tables mbio.03407-25-s0005.pdf Tables S1 and S2.

ASM does not own the copyrights to Supplemental Material that may be linked to, or accessed through, an article. The authors have granted ASM a non-exclusive, world-wide license to publish the Supplemental Material files. Please contact the corresponding author directly for reuse.
