# Supplementary figures and images for: An atlas of novel microtubule-associated proteins in the malaria parasite Plasmodium falciparum
Source: mBio. 2025 Dec 8;17(1):e03407-25. doi: 10.1128/mbio.03407-25 (PMC12802302; doi:10.1128/mbio.03407-25)

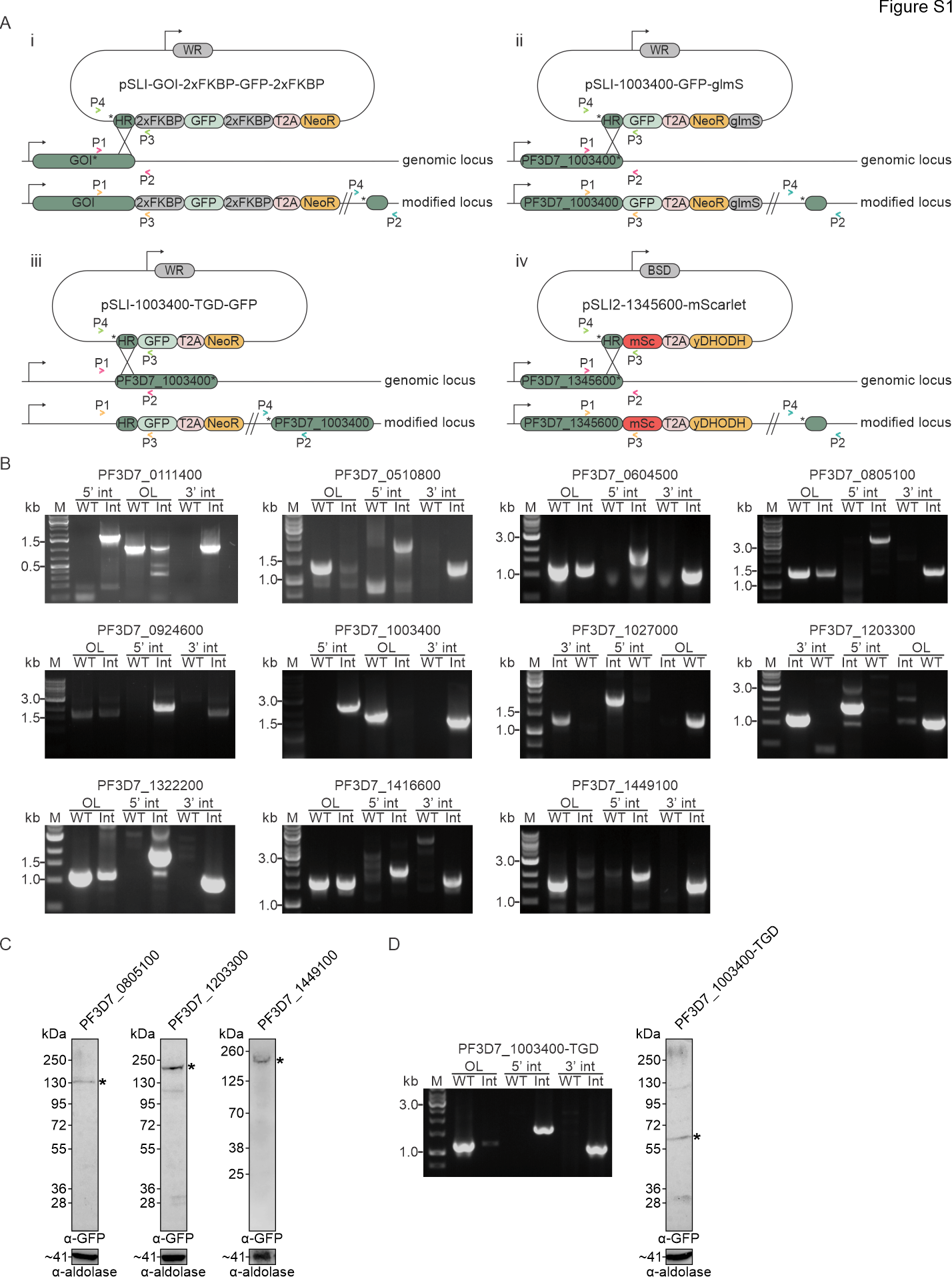

Supplement: Fig. S1 — Additional data for the selected proteins identified in the PfSPM3 BioID. [file mbio.03407-25-s0001.tif]

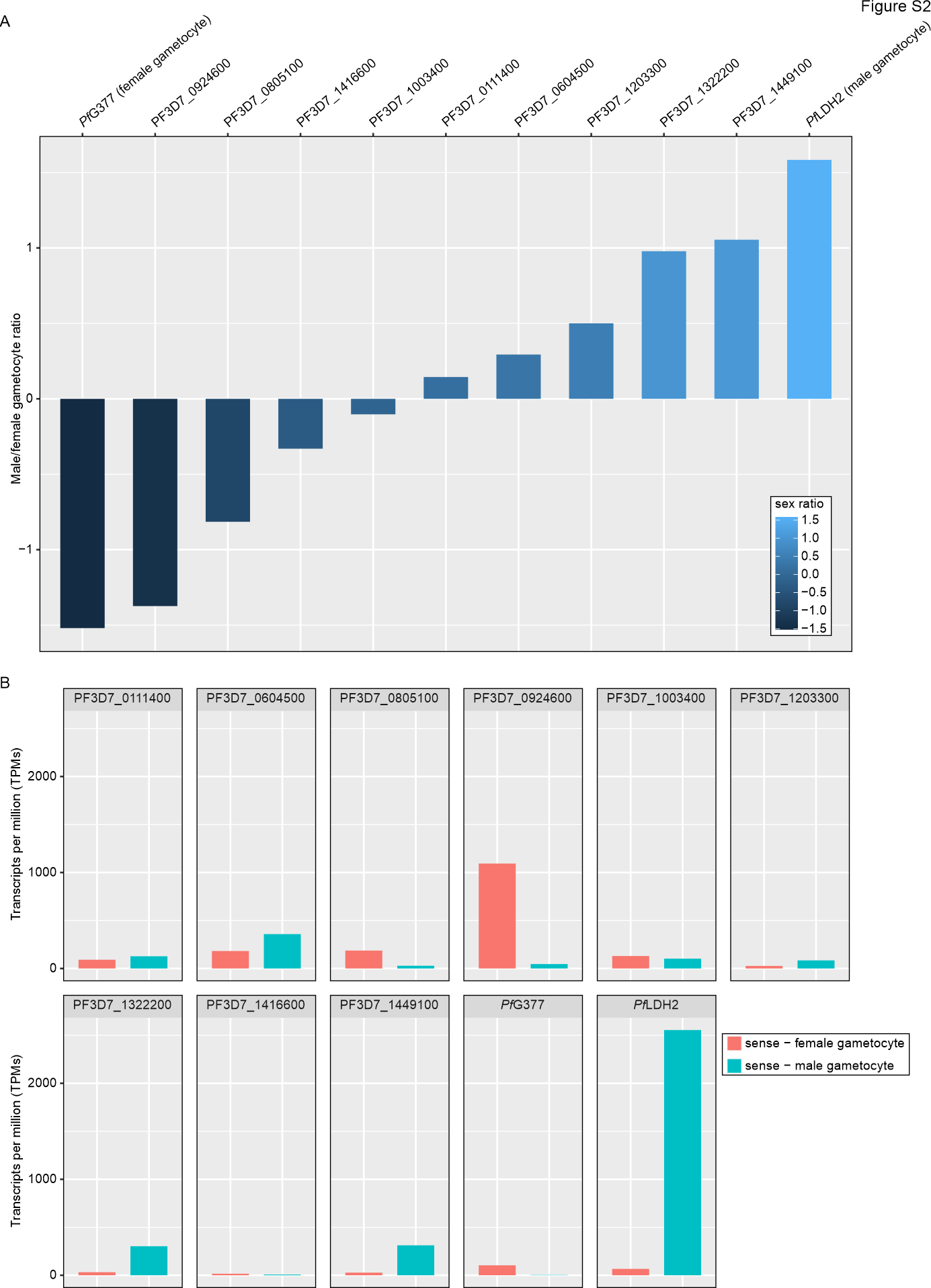

Supplement: Fig. S2 — RNA seq expression of candidates in female and male gametocytes. [file mbio.03407-25-s0002.tif]

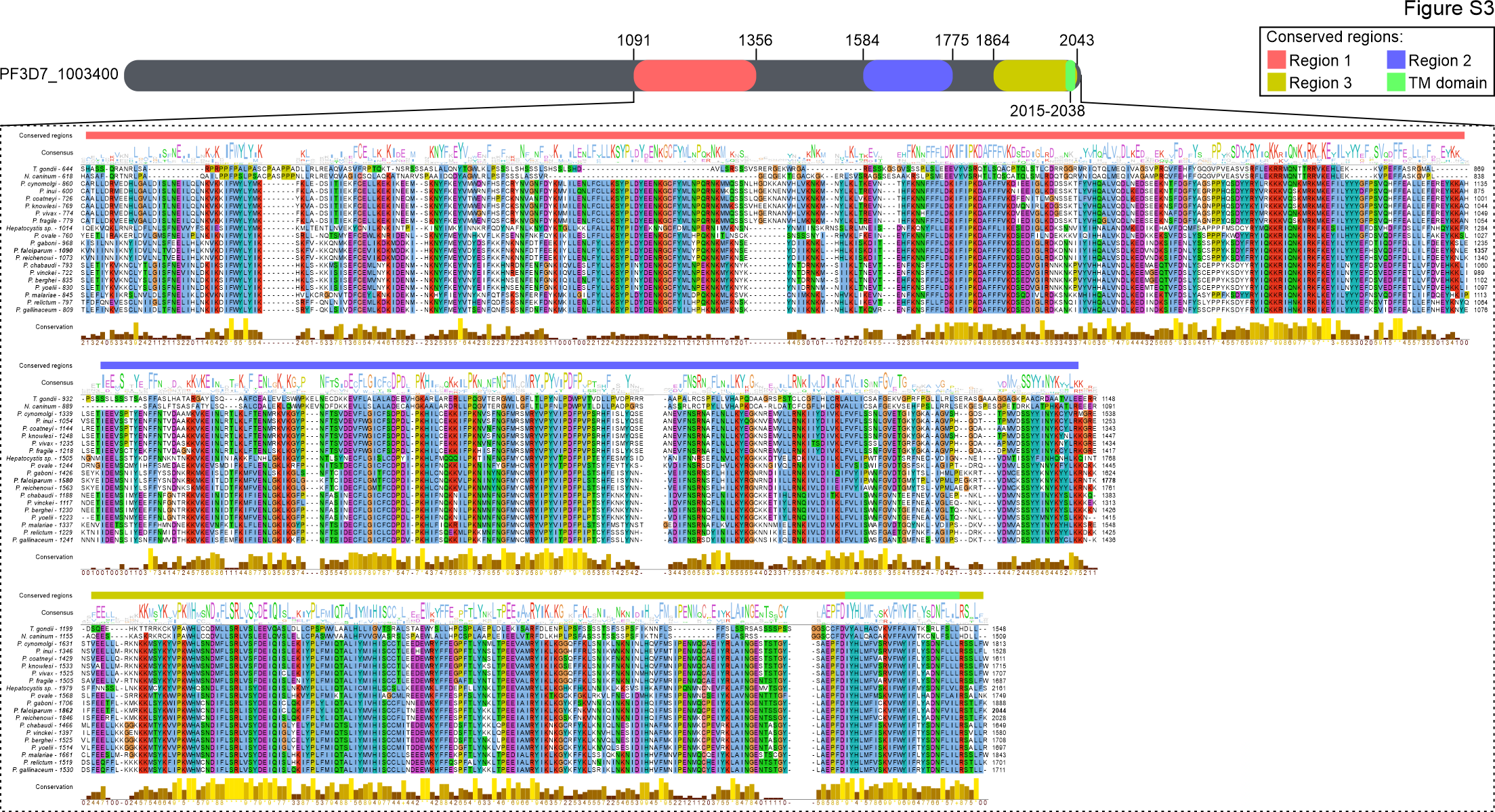

Supplement: Fig. S3 — Schematic representation of the suture-like protein PF3D7_1003400 and sequence alignment with different orthologs. [file mbio.03407-25-s0003.tif]
