# Supplementary material for: An atlas of novel microtubule-associated proteins in the malaria parasite Plasmodium falciparum
Source: mBio. 2025 Dec 8;17(1):e03407-25. doi: 10.1128/mbio.03407-25 (PMC12802302; doi:10.1128/mbio.03407-25)
Supplement: Legends — for supplemental files. [file mbio.03407-25-s0004.pdf]

SUPPLEMENTARY FIGURE LEGENDS

Figure S1. Additional data for the selected proteins identified in the *Pf*SPM3 BioID. (A) Schematic of SLI (i), SLI-gmlS (ii), SLI-TGD (iii) and SLI2 (iv) strategies used to modify the genome of the parasite to produce C-terminally tagged versions of the candidates. HR, homology region with an additional stop codon (*); GOI, gen of interest; GFP, green fluorescent protein; T2A, skip peptide; NeoR, Neomycin resistance cassette; WR, human dihydrofolate dehydrogenase (hDHFR); angle arrows, promoters. P1-P4 arrows, primers for PCR to assess correct genomic integration of the SLI plasmids in (B). **(B)** Diagnostic PCR with genomic DNA isolated from selected proteins or parental iGP-NF54 (WT) parasites. Primer combinations: 5’ integration (P1+P3); 3’ integration (P4+P2) or original locus (OL) (P1+P2). Position of primers as indicated in (A). **(C)** Western Blots of the candidates PF3D7_0805100, PF3D7_1203300 and PF3D7_1449100. **(D)** Diagnostic PCR and Western Blot of the TGD version of PF3D7_1003400. GFP-tagged proteins were detected using mouse anti-GFP antibodies. Aldolase, detected using rabbit anti-aldolase antibodies, was used as a loading control. Asterisks (*****) indicate the expected sizes of the GFP-tagged proteins by Western Blot.

Figure S2. RNA seq expression of the candidates PF3D7_0111400, PF3D7_0604500, PF3D7_0805100, PF3D7_0924600, PF3D7_1003400, PF3D7_1203300, PF3D7_1322200, PF3D7_1416600 and PF3D7_1449100 in female and male gametocytes. (A) Male/female gametocyte ratio of the candidates compared to the female (*Pf*377, in negative value range) and male (*Pf*LDH2, in positive value range) gametocyte markers. Scale of sex ratio was ranked from negative (*Pf*377, female marker) to positive (PfLDH2, male marker) values. **(B)** Male/female gametocyte ratio for each candidate. TPMs, transcripts per million.

Figure S3. Schematic representation of the suture-like protein PF3D7_1003400 and sequence alignment with different orthologs. Orthologs of PF3D7_1003400 were taken from Figure 1 based on the searching of sequences in PlasmoDB and OrthoMCL. ClustalOmega was performed with 19 different proteins. *Cyclospora cayetanensis* ortholog (cyc_05309) was excluded for further analysis as the length of its sequence was less than 1000 aa. On the top of sequence alignment, conserved regions and TM domain were colored. Consensus (on top) and conservation (on bottom) are also shown.

Supplementary Table S1: List of the 181 enriched proteins from the *Pf*SPM3-BioID gametocytes.

Supplementary Table S2: Oligonucleotides used in this study.
