# Supplementary material for: An atlas of novel microtubule-associated proteins in the malaria parasite Plasmodium falciparum
Source: mBio. 2025 Dec 8;17(1):e03407-25. doi: 10.1128/mbio.03407-25 (PMC12802302; doi:10.1128/mbio.03407-25)
Supplement: Supplemental Tables — Tables S1 and S2. [file mbio.03407-25-s0005.pdf]

**Supplementary Table 1. List of the 181 enriched proteins from the *Pf*SPM3-BioID gametocytes**		
Gene ID	Log2 (FC)	Minus log10 (Pvalue)
PF3D7_0419600	2.849815062	4.110698297
PF3D7_1327300	2.680733813	4.155522824
PF3D7_1128200	2.351954745	3.73225585
PF3D7_1027000	2.278936651	3.460274201
PF3D7_1449100	2.274499204	3.79290446
PF3D7_1142100	2.218409249	3.592298147
PF3D7_0510800	2.200272785	3.635865677
PF3D7_1423700	2.182881089	3.915623937
PF3D7_0604500	2.159439166	3.919088646
PF3D7_1003400	2.119250084	3.408559167
PF3D7_0928100	2.07680076	3.168540409
PF3D7_0307300	2.073779463	3.279043155
PF3D7_1356800	2.005610273	3.343758016
PF3D7_0111000	1.996894546	3.782318694
PF3D7_1245100	1.990393417	3.699056872
PF3D7_0927200	1.930541245	3.609280328
PF3D7_1244600	1.919321054	2.069055551
PF3D7_0805100	1.906022038	3.170170245
PF3D7_1317300	1.858674189	2.577122039
PF3D7_1416600	1.855115706	3.436125143
PF3D7_1423500	1.821871936	2.416267474
PF3D7_0919900	1.818783325	2.85857594
PF3D7_0111400	1.812999059	3.539443415
PF3D7_0924600	1.810303247	3.567372189
PF3D7_0320400	1.80979222	1.727797614
PF3D7_0709400	1.803082358	2.292128647
PF3D7_1322200	1.785191803	3.093153912
PF3D7_0105700	1.763059679	3.572853215
PF3D7_1203300	1.762528917	3.507196323
PF3D7_0518700	1.75267028	2.640188224
PF3D7_1408800	1.746777106	3.48627247

PF3D7_1110500	1.742294941	3.039908504
PF3D7_1224300	1.740668569	2.603943523
PF3D7_0706500	1.732732712	3.540033357
PF3D7_1114900	1.710472164	3.350096841
PF3D7_0930400.2	1.706238056	2.554730597
PF3D7_1036900	1.68016684	2.72700475
Pf3D7_1416200	1.670856276	3.2274612
PF3D7_0311400	1.66405619	2.950634265
PF3D7_1420600	1.657939604	2.604076317
PF3D7_0909500	1.65535855	2.544872407
PF3D7_0729100	1.649811529	2.76586631
PF3D7_1325400	1.646009697	3.133726251
PF3D7_0205100	1.645204932	2.885788136
VPS26	1.641404299	3.323994492
PF3D7_1302500	1.639548739	3.337541557
PF3D7_0513600	1.639317662	2.6088508
PF3D7_0704000	1.632576697	3.217708011
PF3D7_0529800	1.612239237	3.135289616
PF3D7_0825900	1.606218715	1.942847595
PF3D7_1319000	1.602713889	3.184140782
PF3D7_1308900	1.600556269	3.287132364
PF3D7_1467600	1.568681925	1.870907545
PF3D7_0111800	1.559155979	1.716800594
PF3D7_0315600	1.558683611	1.720632923
PF3D7_1417100	1.53453363	3.225088334
PF3D7_1420200	1.529030589	3.189343479
PF3D7_1303800	1.527760865	3.123277124
PF3D7_0522100	1.524865671	3.136946724
PF3D7_0916000	1.520280465	2.781410146
PF3D7_0703500	1.517593625	3.275098542
PF3D7_1115200	1.508804098	3.080566769
PF3D7_1019100	1.505972221	3.175646986
PF3D7_1028500	1.490740039	2.953317189
PF3D7_0906400	1.48725809	3.122214141

PF3D7_0318400	1.48212193	2.800426091
PF3D7_0526200.2	1.474020985	2.632704847
PF3D7_1442400	1.470686364	2.626460196
PF3D7_1110400	1.46571565	3.089487117
PF3D7_1103800	1.462779609	3.013256026
PF3D7_0933900	1.459397754	2.434541096
PF3D7_1021200	1.448818106	1.849893278
PF3D7_1006700	1.445265845	2.70479478
PF07_0021	1.44318999	3.115545071
PF3D7_1442700	1.43708226	2.087425229
PF07_0086	1.432024381	1.526123639
PF3D7_1202600	1.404450295	3.068671318
PF3D7_1458500	1.401349007	2.33056847
PF3D7_1212000	1.391634441	2.702514374
PF3D7_1205500	1.38757552	2.83927403
PF3D7_1005600	1.38580487	2.7224435
PF3D7_1356400	1.377403893	3.017526143
PF3D7_1146800	1.376298161	2.484984869
PF3D7_1107300	1.372381059	2.876148686
PF3D7_1140500	1.362020218	3.004720722
PF3D7_0209500	1.355751359	1.431619008
PF3D7_0911200	1.348653628	2.738383415
PF3D7_1219000	1.340812725	2.698703863
PF3D7_1415400	1.337874185	3.012817895
PF3D7_1435600	1.334335228	2.415809039
PF3D7_1229300	1.326356304	1.06905966
PF3D7_1405100	1.321841463	2.050364028
PF3D7_0811500	1.304783918	2.922730958
PF3D7_0218600	1.294083912	1.825542877
PF3D7_1311100	1.291550709	2.86566317
PF3D7_0815200	1.290986739	2.977592664
PF3D7_0522900	1.290076894	2.107659089
PF3D7_1417200	1.286639628	2.883505161
PF3D7_0408200	1.283739436	2.623201755

PF3D7_1138500	1.280782853	2.739564864
PF3D7_0718000	1.280449666	1.677292977
PF3D7_1003700	1.279914763	1.922609817
PF3D7_1336800	1.279028661	2.80970781
PF3D7_1013900	1.264295887	2.594656031
PF3D7_1454300	1.26198652	2.663323173
PF3D7_1466200	1.259335069	2.318988366
PF3D7_0504800	1.257349426	2.44894202
PF3D7_0703000	1.254491521	2.84560981
PF3D7_0303200	1.247157945	2.75446019
PF3D7_1119900	1.246033573	2.520855887
PF3D7_0726600	1.234405741	2.467424597
PF3D7_0612200	1.230804909	1.845090813
PF3D7_1309500	1.228540721	2.834317445
PF3D7_1421000	1.214252778	2.795490413
PF3D7_0823500	1.212861903	1.8580982
PF3D7_1121100	1.207024512	2.643608877
PF3D7_0505900	1.204217866	2.303532125
PF3D7_1233200.1	1.198432004	2.835156015
PF3D7_1362700	1.189008544	2.201136954
PF3D7_1340500	1.187067085	1.766349766
PF3D7_0322200	1.183099237	2.343347923
SET1	1.179692207	1.073446833
PF3D7_0619000.2	1.178260723	2.726105579
PF3D7_0303500	1.176316186	2.805301989
PF3D7_1320700	1.168051687	1.520503452
PF3D7_1019000	1.16795756	2.652228693
PF3D7_1457100	1.166994147	2.474586135
PF3D7_0405700	1.162057065	2.553415927
PF3D7_0508100	1.156549906	2.651909221
PF3D7_1461800	1.154740537	1.694659295
PF3D7_1027700.2	1.153287883	2.699891251
PF3D7_1236100	1.151118537	2.00426664
PF3D7_1025900	1.144524371	2.41491857

PF3D7_1325900	1.144451093	2.721339897
PF3D7_1323800	1.132980838	2.713948372
PF3D7_1402100	1.121672737	2.375527929
PF3D7_0727000	1.108541821	2.379521227
PF3D7_1130700	1.108040875	2.4101596
PF3D7_1423400	1.105447474	2.677071317
PF3D7_0914900	1.097995842	2.291861015
PF3D7_0214300	1.094975863	1.767895056
PF3D7_0103100	1.094150914	2.543014552
PF3D7_1430800	1.087554327	2.422735632
PF3D7_1117900	1.081331743	2.273092429
PF3D7_1234100	1.078414089	2.139476095
eIK1	1.071966296	2.116196548
PF3D7_0626600	1.070271148	1.517170462
PF3D7_1122500	1.068435794	2.225009802
PF3D7_0804900	1.068369493	2.599267269
PF3D7_1438600	1.06628148	1.493860158
PF3D7_1015400	1.065335133	2.52138647
PF3D7_0811300	1.057934795	1.904382005
PF3D7_0602000	1.056167592	1.76100526
PF11_0207	1.055362119	2.610438622
PF3D7_1416900	1.05339637	2.177110506
PF3D7_1021900	1.05326255	1.52858839
PF3D7_0519500	1.052065499	2.557114974
PFD0685c	1.04978536	2.470004814
PF3D7_1344100	1.047118594	2.193303794
PF3D7_0216500	1.042423486	2.431683497
PF3D7_0726500	1.041383115	2.413250484
PF3D7_1011800	1.039027285	2.540083057
PF3D7_0718500	1.038190967	2.33719421
PF3D7_1134300	1.037215354	2.532248238
PF3D7_1471600	1.037085262	2.382062322
PF3D7_0409000	1.031313353	2.202885009
PF3D7_0907000	1.027186479	1.28380732

PF3D7_0611800	1.025756271	2.533954908
PF3D7_1308700	1.02115403	1.76034397
PF3D7_0110500	1.018745532	2.054701177
PF3D7_0507800	1.018451077	2.169925412
PF3D7_1358600	1.017671334	2.435658654
PF3D7_0307700	1.017048751	2.519788613
PF3D7_0814200	1.016171982	0.903450796
PF3D7_0919800	1.015252718	2.249784525
PF3D7_0505800	1.01283552	2.163390268
PF3D7_1241400	1.007743408	1.854727902
PF3D7_1212700	1.00623858	1.457224675
PF3D7_0522200	1.006128874	2.096677294
PF3D7_1227700	1.002951615	1.668308502
MAL7P1.13	1.002143752	2.110427494

Supplementary Table S2. Oligonucleotides used in this study	
Primer name	DNA sequence
0111400 Tag fwd	atactcgcggccgcTAAATGAATCCATATCATCATAGTAGTCGTAGCG
0111400 Tag rev	ccatacgcgtcctaggATTGCAAAATGCTTGGTCATAAAACTTTTGACC
0604500 Tag fwd	ccaagctatttaggtgacactatagaatactcgcggccgctaaGATGTTGCAGACTTTCCATATTTAACAACAGGTG
0604500 Tag rev	CCACCAGCACCAGCAGCAGCAGATCTTGATCTCAATCCTGAcctaggTTTTTTTTTTTTTTCATTATTATTTGTATTATTTTTATTGTTTATTCTG
0805100 Tag fwd	ccaagctatttaggtgacactatagaatactcgcggccgctaaAGTAGCACATTTATTCCCTTACCACATGC
0805100 Tag rev	CCAGCACCAGCAGCAGCAGATCTTGATCTCAATCCTGAcctaggAAAATTAAAGAATTTAACTCCCCTTTTTTTTAACATATTATCATTTTCTTCC
0924600 Tag fwd	ccaagctatttaggtgacactatagaatactcgcggccgctaaACTTTGGATGAGCGTTATACTCCTTTTCG
0924600 Tag rev	CCACCAGCACCAGCAGCAGCAGATCTTGATCTCAATCCTGAcctaggGATGAGTAGATGCTCATCCATTTTTTTTTTTATCTTATCG
1003400 Tag fwd	cgccaagctatttaggtgacactatagaatactcgcggccgctaaGGAATGTATACTCCATTGGTTCCTATGC
1003400 Tag rev	gccatgttgtcttcttctcccttacttaccatacgcgtcctaggTTTTTTTGTTGGTTTGTTTTTAAATAAGGTTGATCG
1203300 Tag fwd	ccaagctatttaggtgacactatagaatactcgcggccgctaaGCATTACCAATGGGTATGGAGAAAG
1203300 Tag rev	CCACCAGCACCAGCAGCAGCAGATCTTGATCTCAATCCTGAcctaggTAACATACTTCCTTCGTGTTCCCTCTG
1322200 Tag fwd	ccaagctatttaggtgacactatagaatactcgcggccgctaaCGTAGAGAATCTCCCAACGTTTCTCAC
1322200 Tag rev	CCACCAGCACCAGCAGCAGCAGATCTTGATCTCAATCCTGAcctaggCTTATTTGTATTTTCTATACAGAAAAAAGAAAAATGAAC
1416600 Tag fwd	ccaagctatttaggtgacactatagaatactcgcggccgctaaCCAAAAATGATTGGTGAAGACAATTCCTATGTC
1416600 Tag rev	CCACCAGCACCAGCAGCAGCAGATCTTGATCTCAATCCTGAcctaggTAAAAATTCTGTAATACTTATGTACCATTTCACTATACC
1449100 Tag fwd	ccaagctatttaggtgacactatagaatactcgcggccgctaaTGGATGATGTAATATTACCATGTTTGAAAAAGGC

1449100 Tag rev	CCACCAGCACCAGCAGCAGCAGATCTTGATCTCAATCCTGAcctaggATTAAATTTAATTTTCCCCAAAACAAGGTCG
1003400 TGD fwd	gaatactcgcggccgcTAATTATATTCGAAGAGTAATTTGAATGTAGGTAACATTCC
1003400 TGD rev	cacctctagcacgcgtTACATCCCATATTAACTCATATTTAACAAATGGATG
0111400 int fwd	CATGTACAAATGATAAGTTTGTAGGTG
0111400 int rev	CATACGTTTGGTTGTATAACGAATG
0604500 int fwd	CGAAAAAATGAAATGGACTATATTAAGTGAGAG
0604500 int rev	GTAAATAAAAGGACGAAGGATAGAG
0805100 int fwd	GTTTCCTTTACTACCTTCTTCGCC
0805100 int rev	CAATTACAATGTATGAACCAACACATAAATATAGAATC
0924600 int fwd	GGAGATGATGGGAAATTGATAAGTAGTTG
0924600 int rev	CCGTAACAGTATTTTGTCGTTTTG
1003400 int fwd	CCATGGAATTTTGGTGTAGACAC
1003400 int rev	CTATTTTGGAATGTTGTGGTTTTTTGGTG
1203300 int fwd	CAATGAAAGCTCCTGCTTATTTTTCTGC
1203300 int rev	CGTATAAAATAAATGAATATACAGGGGCATACTC
1322200 int fwd	GTAACGAATCAGCCAGTTCTGTTC
1322200 int rev	GGGTCACCCGTCAACACAATATTC
1416600 int fwd	GAAAGTATCTACACCTTTAAGTGATGTACC
1416600 int rev	GAGACACACAACATGTCGATTTAGAAG
1449100 int fwd	GCTATTACAAAAGATCATGAATGGACAAAAC
1449100 int rev	CAGCCAAACATGAGCATATTTCATGC
3’ Int fwd	GCGGATAACAATTTCACACAGG
GFP-seq rev	GAATTGGGACAACTCCAGTG
1003400 TGD int fwd	GGATCTACTTATAAGAACTATTCAGTAGTACC
1003400 TGD int rev	GAGGTATTGTTCTTTTCTGAAGACTG
Neo-seq rev	GAGAACCTGCGTGCAATCC
1345600 5' int fwd	CACTAAAATATTATTTATATGTG
1345600 3' int rev	GTAATACATCTATAATTATATATATG
pARL_sense_55 fw	GGAATTGTGAGCGGATAACAATTTCACACAGG
mScarlet rv	GGTCACGAGTTTGAGATTGAGGG
